# Novel homozygous *RARS2* mutation in two siblings without pontocerebellar hypoplasia – further expansion of the phenotypic spectrum

**DOI:** 10.1186/s13023-016-0525-9

**Published:** 2016-10-21

**Authors:** S. Lühl, H. Bode, W. Schlötzer, M. Bartsakoulia, R. Horvath, A. Abicht, M. Stenzel, J. Kirschner, S. C. Grünert

**Affiliations:** 1 Department of Pediatrics and Adolescent Medicine, University Medical Center, Ulm, Germany; 2 Department of Diagnostic and Interventional Radiology, Section Neuroradiology, University Medical Center, Ulm, Germany; 3 John Walton Muscular Dystrophy Research Centre, Institute of Genetic Medicine, Newcastle University, Newcastle upon Tyne, UK; 4 Medical Genetics Centre, Munich, Germany; 5 Department of Pediatric Radiology, Kliniken der Stadt Köln, Köln, Germany; 6 Department of Neuropediatrics and Muscle Disorders, Medical Center – University of Freiburg, Faculty of Medicine, Freiburg, Germany; 7 Department of General Pediatrics, Adolescent Medicine and Neonatology, Medical Center – University of Freiburg, Faculty of Medicine, Freiburg, Germany

**Keywords:** Mitochondrial disease, RARS2, Pontocerebellar hypoplasia, OXPHOS, Mitochondrial arginyl transfer RNA synthetase

## Abstract

**Background:**

Pontocerebellar hypoplasia type 6 (PCH6) is a mitochondrial disease caused by mutations in the *RARS2* gene. *RARS2* encodes mitochondrial arginyl transfer RNA synthetase, an enzyme involved in mitochondrial protein translation. A total of 27 patients from 14 families have been reported so far. Characteristic clinical features comprise neonatal lactic acidosis, severe encephalopathy, intractable seizures, feeding problems and profound developmental delay. Most patients show typical neuroradiologic abnormalities including cerebellar hypoplasia and progressive pontocerebellar atrophy.

**Methods:**

We describe the clinical, biochemical and molecular features of 2 siblings with a novel homozygous mutation in *RARS2*. Both patients presented neonatally with lactic acidosis. While the older sibling had severe neurological symptoms with microcephaly, seizures and developmental delay, the younger patient was still neurologically asymptomatic at the age of 2 months.

**Results:**

MRI studies in both children lacked pontocerebellar involvement. The expression of the OXPHOS complex proteins was decreased in both patients, whereas oxygen consumption was increased.

**Conclusions:**

Characteristic neuroradiological abnormalities of PCH6 such as vermis and cerebellar hypoplasia and progressive pontocerebellar atrophy may be missing in patients with *RARS2* mutations. *RARS2* testing should therefore also be performed in patients without pontocerebellar hypoplasia but otherwise typical clinical symptoms.

## Background

Pontocerebellar hypoplasia type 6 (PCH6) is a mitochondrial disease with autosomal recessive inheritance caused by mutations in the *RARS2* gene. *RARS2* encodes the mitochondrial arginyl transfer RNA (tRNA) synthetase, an enzyme which belongs to the group of mitochondrial aminoacyl tRNA synthetases. These nuclear encoded mitochondrial proteins play a key role in mitochondrial protein translation by catalyzing the attachment of amino acids to their cognate tRNA molecules [1]. Defects of mitochondrial aminoacyl tRNA synthetases have emerged as an important cause of perinatal or infantile onset respiratory chain disorders with often early fatal outcome [1]. Mutations in *RARS2* were first described in a consanguineous Sephardic Jewish family [2]. Following the original description, a total of 27 patients from 14 families have been reported. The typical clinical picture of PCH6 comprises neonatal lactic acidosis, severe encephalopathy, intractable seizures, hypotonia, spastic quadriplegia, microcephaly, feeding problems and profound developmental delay [2, 3]. Most patients present characteristic neuroradiological abnormalities including cerebellar hypoplasia and progressive cerebral cortical atrophy together with progressive pontocerebellar atrophy [3]. *RARS2* mutations are associated with variable oxidative phosphorylation defects.

We describe the clinical, biochemical and molecular features of 2 siblings with a novel homozygous mutation in *RARS2*. MRI studies in both children lacked pontocerebellar involvement, and the younger patient was still neurologically asymptomatic at the age of 2 months, expanding the clinical picture of this disease. A defect of mitochondrial translation was confirmed in fibroblasts of both patients. Our cases demonstrate that pontocerebellar hypoplasia (PCH) is no sine qua non for the diagnosis of *RARS2* mutations.

## Methods

### Patient 1

**Fig. 1:**
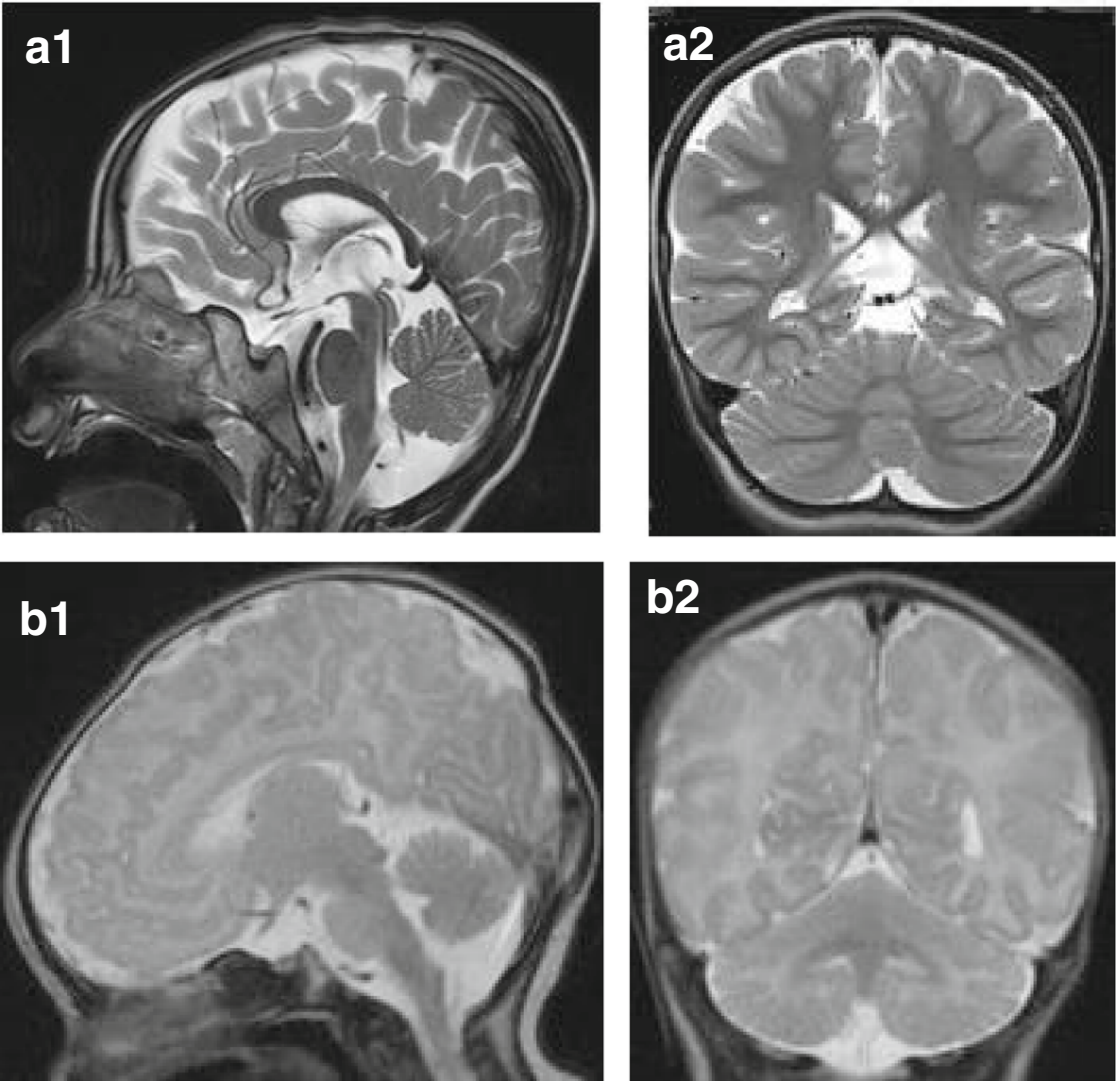
Brain MRI T2-weighted, sagittal (1) and coronal (2) sections. **a** *Patient 1*, microcephaly, mild enlargement of the subarachnoid space and atrophy of the white matter, but no pontocerebellar hypoplasia at age 40 months. **b** *Patient 2*, normal MRI at age 10 days

Sibling 1 is the second son of healthy consanguineous Saudi Arabian parents (first-degree cousins) with no family history of metabolic disorders. He was born at term (birth weight 3100 g, 25th–50th percentile). On the first day of life he became lethargic and diagnostic work-up revealed metabolic acidosis with mildly elevated lactate. On day 2 he showed hypoglycemia and signs of infection. At the age of 3 months he developed muscular hypotonia and convulsions. Under vigabatrin therapy seizure-frequency decreased. At the age of 5 months convulsion-pattern changed and the EEG showed hypsarrythmia as well as a burst suppression pattern. Consequently, pyridoxine, folinic acid, biotin and steroids were applied, further improving frequency and duration of seizures. At the age of 1, marked motor retardation was evident. On valproic acid and clobazam supplementation the patient had only rare seizures since the age of 34 months. At 40 months he was first examined in Germany. A brain MRI at that age showed mild enlargement of the subarachnoid space, atrophy of both thalami, the mammillary bodies and of the white matter, but no signs of PCH (Fig. 1a). A first MRI in Saudi Arabia at the age of 3 months had been normal, a second one at the age of 25 months had shown nearly the same pathologies as the one in Germany at the age of 40 months. Muscle biopsy at the same time showed normal histology. Assessment of respiratory chain enzyme activities revealed mild reduction of the activity of some complexes (Table 1). The electromyography was normal. Ophthalmological examination did not reveal any pathology. Neurotransmitters in CSF were normal. Comparative genomic hybridization (CGH) detected no abnormalities. A next-generation-sequencing panel including 23 nuclear encoded genes involved in mitochondrial translation yielded a novel homozygous sequence variant, c.392T > G; p.(Phe131Cys) in the *RARS2* gene. This mutation was confirmed by Sanger sequencing. Both parents were found to be heterozygous for the variant. At the age of 45 months muscular hypotonia, severe mental and motor retardation, cerebral visual impairment, microcephaly and symptomatic epilepsy with continuous spikes and waves during slow-wave sleep (csws) were seen. Valproic acid was replaced by Sultiam and methylprednisolone pulse therapy was performed, which resulted in improvement of the EEG and freedom from seizures at that time.

**Table 1:** Enzyme activities of the respiratory chain measured in muscle homogenate of patient 1

[mUnit/mg protein]		Reference range	[mUnit/mUnit CS]		Reference range
Citrate synthase (CS)	168	150–338			
Complex I	**23**	28–76	C1/CS	0.14	0.14–0.35
Complex I + III (C13)	**42**	49–218	C13/CS	0.25	0.24–0.81
Complex II (C2)	32	33–102	C2/CS	0.19	0.18–0.41
Complex II + III (C23)	**45**	65–180	C23/CS	**0.27**	0.30–0.67
Complex III (C3)	327	304–896	C3/CS	1.95	1.45–3.76
Cytochrome oxidase (COX)	201	181–593	COX/CS	1.20	0.91–2.24
Complex V (C5)	**69**	86–257	C5/CS	**0.41**	0.42–1.26
Pyruvate dehydrogenase (PDHC)	**3.9**	5.3–19.8	PDHC/CS	**0.023**	0.026–0.079

Some enzymes of the respiratory chain showed mildly reduced activities (bold data﻿). However, in relation to the activity of citrate synthase the impairment was only minimal

### Patient 2

**Fig. 2:**
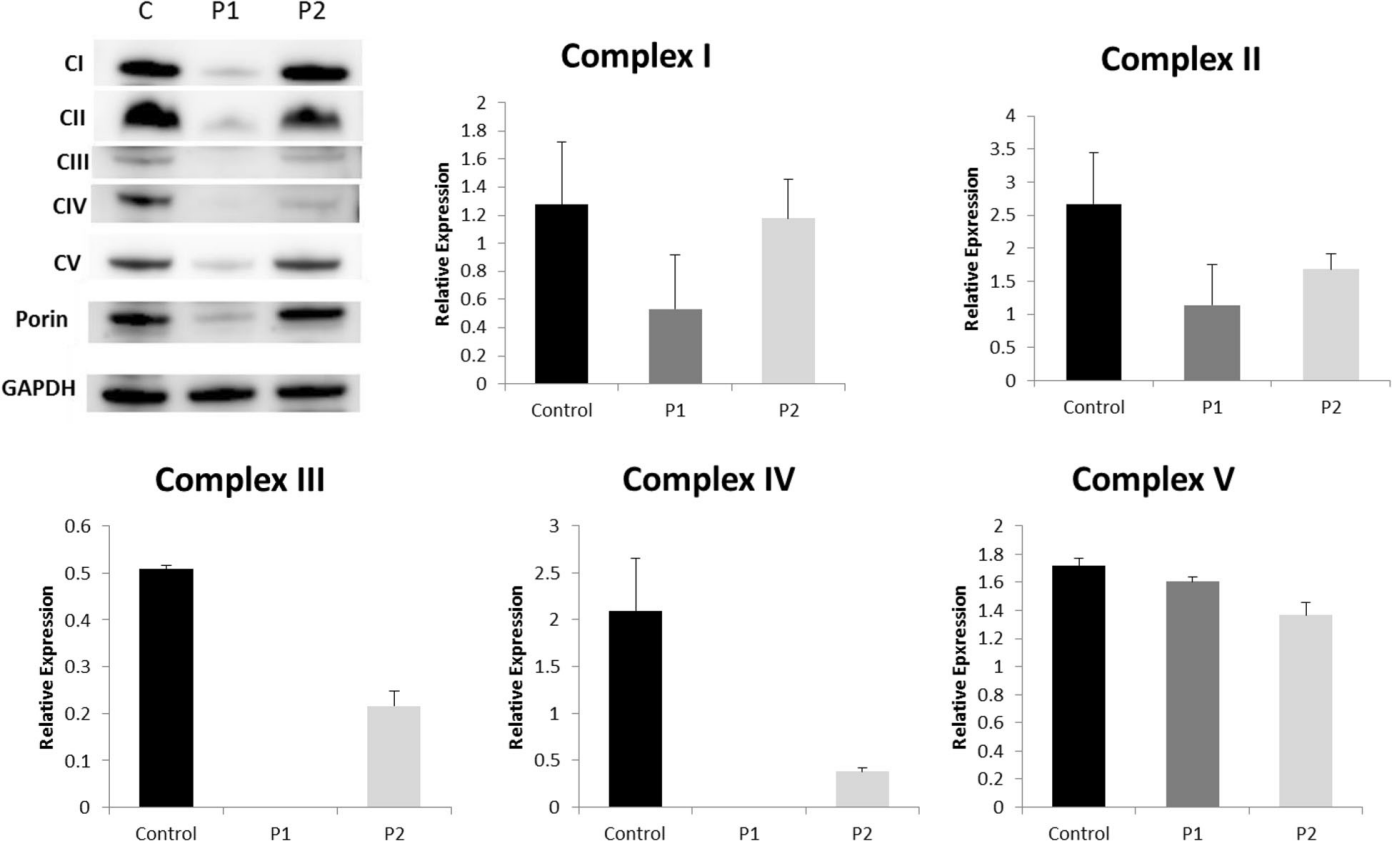
SDS-PAGE for mitochondrial proteins of the control and 2 patients’ (P1, P2) fibroblast cell lines. P1 and P2 presented decreased levels of protein expression of the OXPHOS complex proteins. While in P1 the expression of all complexes was severely impaired, in P2 the defect was less severe and not all complexes were affected

**Fig. 3:**
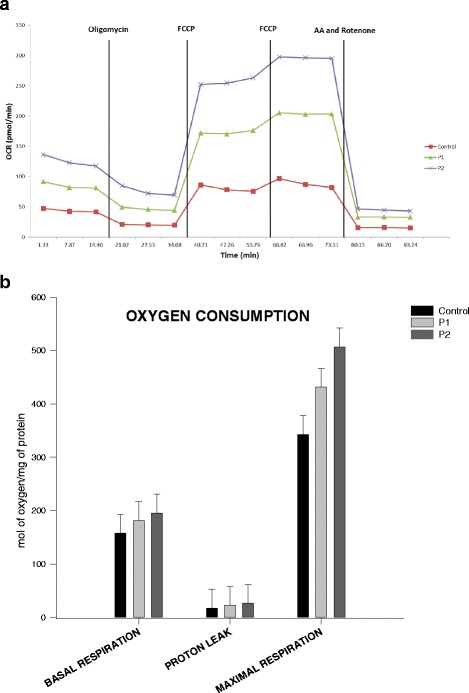
**a**/**b** Oxygen consumption measurement with Seahorse assay. P1 and P2 illustrated increased levels of oxygen consumption, suggesting compensatory changes

The girl is the younger sister of patient 1. She was born at term (birth weight of 2810 g, 10th–25th percentile; length 49 cm, 25th–50th percentile; head circumference 34 cm, 25th–50th percentile) and showed good postnatal adaption (Apgar 10/10 at 5 and 10 min, respectively, pH of cord blood 7.31). On day two she became tachypnoeic. Blood gas analysis revealed a severe lactic acidosis (pH 7.22, bicarbonate -18 mmol/l, pCO2 20 mmHg, lactate of 22 mmol/l) and hypoglycemia (1.6 mmol/l). The ammonia concentration in plasma was normal (78 μmol/l), prothrombin time was 27 % and partial thromboplastin time 56 s. There were no signs of neonatal infection (CRP < 3 mg/l). She received a 10 % dextrose infusion, buffering with sodium bicarbonate, fresh frozen plasma and vitamin K. Because a metabolic disorder was suspected she was transferred to the intensive care unit of our metabolic centre. At arrival she was in a stable clinical condition without neurological symptoms. Due to suspected mitochondrial disease, a low-glucose high-fat infusion (5 g/kg body weight/day and 3 g/kg body weight/day, respectively) was begun and buffering with sodium bicarbonate was continued. She was also started on carnitine (300 mg/d), thiamine (200 mg/d), riboflavin (100 mg/d) and coenzyme Q10 (50 mg/d). Within 12 h the lactate concentration normalized (2.0 mmol/l). On day 3, enteral feeding was initiated. Initially, the girl received a high-fat diet (50 % breast milk, 50 % KetoCal 4:1), however, as lactate levels remained below 5 mmol/l, the child could be fully breast-fed. At this point, the results of the genetic panel diagnostics for mitochondrial translation defects of the older brother became available. As the clinical picture was well compatible with a *RARS2* defect genetic analysis of the *RARS2* gene was performed and revealed the same homozygous mutation (c.392T > G; p.Phe131Cys) as found in her brother. At day 5 an EEG was perfomed which yielded unremarkable results. A brain MRI on day 10 showed no abnormalities, especially no signs of PCH or cortical/subcortical atrophy (Fig. 1b). The metabolic condition remained stable with lactate concentrations between 1.5–4.0 mmol/l, and the girl was dismissed from hospital on day 18 in good clinical condition without any neurological abnormalities. The supplementation of coenzyme Q10, riboflavin and thiamin was continued, the carnitin administration was stopped. During the following weeks she showed normal weight gain and stayed neurologically asymptomatic until age 2 months when the family returned to Saudi Arabia. The results of RC enzyme expression in fibroblasts as well as the assessment of oxygen consumption in fibroblasts are shown in Figs. 2 and 3.

### Genetic studies

Next-Generation Sequencing to detect mutations in all coding exons as well as their flanking intronic regions using DNA-targeted enrichment (SureSelect XT Target Enrichment; IlluminaR sequencing technology) was performed. Bioinformatic analysis of collected sequencing data was performed by means of BWA Version 0.7.8-r455, SAMtools Version 0.1.19-44428cd, snpEff Version 3.3f und Alamut-HT Version 1.1.8. Reference sequence: GRCh37/hg19 assembly. The nomenclature of sequence variants is in accordance with HGVS recommendations [4].

### Cell culture

Fibroblasts were obtained from the Newcastle Biobank at the Institute of Genetic Medicine, Newcastle University. Informed consent was obtained from all subjects. Fibroblasts were grown in high glucose Dulbeccos modified Eagle’s medium (Sigma, Poole, UK) supplemented with 10 % fetal bovine serum.

### Oxygen consumption

Oxygen consumption was measured in adherent fibroblasts with a XF96 Extracellular Flux Analyzer (Seahorse Bioscience Billerica, MA, USA) as described previously [5]. Each cell line was seeded in 12 wells of a XF96-well cell culture mircoplate (Seahorse Bioscience) at 30x10^3^ cells/well in 80 μL of DMEM, and incubated for 24 h at 37 °C in a 5 % CO_2_ atmosphere. After replacing the growth medium with 180uL of bicarbonate-free DMEM pre-warmed at 37 °C, cells were pre-incubated for 30 min before starting the assay procedure. Basal respiration, proton leak, maximal capacity respiration and non-electron transport chain respiration were determined by adding 1 μM oligomycin, carbonyl cyanide-ptrifluoromethoxyphenylhydrazone (FCCP) (2 injections of 0.5 μM and 1 μM, respectively) and 1 μM Rotenone/Antimycin, respectively. The data were corrected by the NMR and expressed as mol of oxygen/mg of protein. The quantity of protein was measured by Bradford method [6].

### SDS-PAGE

Cells were trypsinised and centrifuged at 1300 rpm for 5 min. The obtained cell pellets were subsequently resuspended in 50ul of lysis buffer (1 M Tris-HCl pH7.5, 5 M NaCl, 1 M MgCl2, 10 % Triton X, Protease Inhibitor (Roche)), vortexed for 30 s every 5 mins (3 times) and subsequently centrifuged at 12.000 rpm for 5 mins. Protein quantity within the remaining supernatant containing the cellular extracts was measured by the Bradford method [6].

NuPAGE™ Novex™ 4–12 % Bis-Tris Protein Gels and NuPAGE® MES SDS Running Buffer (Thermo Fisher Scientific) were utilized for the pre-cast gel and running buffer. Electrophoresis and sample preparation were performed according to manufacturer’s instructions. Protein samples with a final concentration of 20ug/ml were loaded to each well, and the iBlot® Dry Blotting System (Thermo Fisher Scientific) was used to transfer the proteins as per the manufacturer’s instructions. Membranes were incubated for 1 h in blocking buffer (5 % non-fat milk in TTBS) at room temperature. Three different primary antibodies were used for detection of the OXPHOS complexes (Abcam, ab110413, 1:250 dilution), GAPDH (Santa Cruz, sc-25778, 1:5000 dilution) and Porin (Abcam, ab15895, 1:1000 dilution) respectively. The incubation with the primary antibodies was held overnight at 4 °C. Clarity™ Western ECL Blotting Substrate (BioRad) and the Amersham Imager 600 (GE Healthcare Life Sciences) were used for high-resolution digital imaging of the protein membranes.

## Results

### Genetic studies

Next-generation sequencing based analysis identified a novel homozygous *RARS2* missense mutation, c.392T > G; p.Phe131Cys, in both siblings. This variant has not been observed in sequence analysis of normal individuals or other patients with suspected PCH6, nor is it represented in the Single Nucleotide Polymorphism (dbSNP) or ExAc data base. The predicted amino acid exchange affects a highly conserved position of the arginyl-tRNA synthetase core domain. Bioinformatic programs including Alamut-HT Version 1.1.8, the Sorting Intolerant from Tolerant (SIFT) and PolyPhen [7, 8] indicated pathogenicity of the amino acid exchange.

### SDS-PAGE

We measured the protein expression levels of the respiratory chain complexes. Both patients showed decreased expression levels of OXPHOS (Fig. 2) compared to the control. The relative expression of porin, a protein located in the outer mitochondrial membrane, is significantly decreased in Patient1 (*p* = 0.0084) compared to the control. As a result, CIII and CIV were not detected due to really low expression levels.

### Oxygen consumption

To investigate the mitochondrial defect of the patients’ cell lines we measured the oxygen consumption levels in primary fibroblasts (Fig. 3). Both patient cell lines showed slightly increased levels of oxygen consumption in terms of basal respiration and maximal respiration. Patient 2 presented higher levels of basal and maximal respiration compared to the control and patient 1. However, none of the differences is statistically significant. Overall, the increased levels of basal and maximal respiration compared to the control, in combination with the decreased protein expression levels of OXPHOS complexes in both patients may imply the presence of a compensatory mechanism in fibroblasts.

## Discussion

PCH6 due to mutations in the *RARS2* gene is a rare mitochondrial translation defect, and only 29 patients from 15 families have been reported so far, including the 2 patients in this report. Giving an overview on the first 11 cases, Cassandrini et al. [3] emphasized the exquisitely similar clinical phenotype of this genetic disorder. All patients presented with comparable neurological symptoms of encephalopathy with intractable seizures and severe developmental delay. As in other mitochondrial diseases, the brain seems to be the most vulnerable organ. This can be explained by the higher request in oxidative substrates of the developing brain compared to other tissues [3]. Other organ manifestations such as cardiac, ocular, renal or hepatic symptoms are no common features of this disease. Dysmorphic features are usually lacking, however, one British girl was described with a progressive encephalopathy with edema, hypsarrhythmia, and optic atrophy (PEHO) -like presentation including edema of the hands, feet and face as well as facial abnormalities [9]. The most severe cases were reported by Lax et al. [1]: Two sisters presented perinatal neurologic features typical of PCH6 accompanied by cardiomyopathy, hydrops and pulmonary hypoplasia and died within the first 2 weeks of life. On the other side of the spectrum, Li et al recently described 2 Hispanic siblings with a rather mild form of the disease due to a mutation in the promoter of the *RARS2* gene [10]. The older one was reported to have had normal development until the age of about 6 months. Similarly, our patient 2 - although she presented with severe lactic acidosis on the second day of life - was still neurologically asymptomatic at the age of 2 months (last follow-up) and therefore displays a rather mild phenotype. The different clinical severity in patient 1 and 2 underlines the fact that the clinical phenotype cannot be fully ascribed to the underlying mutations and their impact on mitochondrial arginyl tRNA synthetase activity and the respiratory chain in muscle or fibroblasts [3, 9, 11]. Phenotypic variability within the same family has already been mentioned in previous reports [10] and may be caused by environmental factors, stochastic events and the genetic background. Different tissue expression of mitochondrial arginyl tRNA synthetases, different vulnerability of certain cells with respect to mitochondrial arginyl tRNA synthetase dysfunction or yet unknown functions of mitochondrial arginyl tRNA synthetases, such as involvement in cell signaling, regulation of transcription and splicing, have also been discussed as possible causes for the clinical heterogeneity [3]. So far, no asymptomatic individuals have been identified yet, i.e. by family screening, however, mild or asymptomatic cases may potentially be underdiagnosed.

The very early onset and severity of symptoms in most affected patients suggests a prenatal onset of the disease. This is also confirmed by neuropathologic data which have been published recently [1]. Post mortem studies of the brain of 2 twin sisters with *RARS2* mutations who died within the first 2 months of life revealed most profound changes in the cerebellum and in cerebellum-associated nuclei. These findings lead to the hypothesis that *RARS2* mutations already have small adverse effects during early embryologic development followed by midgestation developmental slowing or cessation and later regression in select anatomic regions [12].

The earliest abnormality in patients with *RARS2* mutations is usually lactic acidosis due to impairment of the respiratory chain. As in our sibling 2, it may be very pronounced or even life-threatening during the neonatal period [1, 9], but usually resolves spontaneously. Detection of lactic acidosis in a newborn can give an important diagnostic hint and may be the only feature suggestive of mitochondrial disorder at this age. This is especially important as lactic acidosis usually becomes less pronounced or even disappears with older age and may be overlooked. Why extremely high lactate levels only occur within the first few days of life remains unclear. Since recurrent metabolic crises during catabolic episodes triggered by infections as seen in other mitochondrial disorders have not yet been described in patients with *RARS2* mutations, the neonatal catabolism is probably not the only reason.

The effects of *RARS2* mutations on the respiratory chain (RC) and the OXPHOS system are variable. While some patients display severe deficiencies of one or more RC complexes [1, 3, 11], others have normal enzyme activities in muscle biopsies [2, 3, 13, 14]. It has been postulated that disordered mitochondrial messenger RNA translation may not be the only mechanism of impairment or that a secondary mechanism may exist to allow some translation [11]. Protein expression of the OXPHOS complex proteins in our patients was studied in fibroblasts and showed decreased expression of multiple RC enzymes with the defect being much more pronounced in patient 1. Interestingly, despite lower protein complex expression in patient cell lines, both basal and maximal respiration were higher in patients compared to controls. These results may imply the presence of a compensatory mechanism in fibroblasts helping them to function properly despite the decreased expression of OXPHOS complexes. Although RC abnormalities in muscle and/or fibroblasts are helpful in the differential diagnosis if present, they are not obligatory, and their absence does not rule out PCH6.

The typical neuroimaging findings described in the first patients identified with *RARS2* mutations lead to the classification of this defect as PCH6. In the majority of patients MR imaging within the first months of life already revealed some affection of the pontocerebellum, ranging from mild vermis cerebellar hypoplasia to profound PCH and gyral immaturity [1, 3, 12]. Normal MRI findings were reported in only three patients within the first 2 ½ months of life [3, 9, 14], however, in one patient MR spectroscopy revealed an increased lactate peak. Two further patients without PCH within the first 4 months of life but otherwise striking abnormalities including marked supratentorial atrophy and subdural effusions have been reported by Kastrissianakis et al. [15]. In both of these siblings cerebellar atrophy occurred within the first year of life. One patient was already examined *in utero* due to an older affected sibling [10]. The prenatal fetal MRI was perfomed at 22 weeks fetal age and demonstrated no brain abnormalities. The typical neuroimaging feature in older patients is progressive cerebral, cerebellar and pontine atrophy, resulting in marked microcephaly. Therefore, repeat imaging might reveal the diagnosis where an initial MRI is apparently normal [9]. In contrast to most other patients reported to date, patient 1 in this study showed no involvement of the pontocerebellum at the age of 40 months despite mild enlargement of the subarachnoid space, atrophy of both thalami, the mammillary bodies and of the white matter, demonstrating that cerebellar hypoplasia and atrophy may be missing in some patients with PCH6. As Cassandrini et al. [3] have already emphasized, the usual lack of basal ganglia involvement is noteworthy for a metabolic encephalopathy related to a defective OXPHOS system. Taken together, neither a normal MRI within the first months of life nor missing pontocerebellar abnormalities within childhood should preclude *RARS2* testing in patients with otherwise suggestive symptoms.

A total of 23 different mutations were reported in the 14 families published to date, including 13 missense mutations, 7 intronic splite site mutations, 2 deletions and one mutation in the promoter of *RARS2*. The only common mutation which has been found in 3 unrelated families in heterozygosity is c.35A > G. Rankin et al. [9] demonstrated that this missense mutation leads to aberrant splicing resulting in insertion of part of intron 1 and generation of a premature termination codon. Of the 27 known cases the majority of 19 patients harbour compound heterozygous mutations while homozygous mutations were only detected in three families [2, 10, 16]. The detection of patients from non-consanguineous families with compound heterozygous *RARS2* mutations lead to the hypothesis that this disorder may be more prevalent than initially recognized [11]. The c.392T > G variant found in the two siblings of this study has not been described before. It affects a highly conserved amino acid within the core domain of the arginyl tRNA synthase. Taking into consideration the clinical presentation of the 2 patients, the biochemical findings and the fact that the mutation segregates with PCH in the family we think that the c.392T > G mutation is pathogenic. In the future, whole exome screening will probably play an increasing role in the diagnosis of neurodevelopmental disorders including metabolic diseases, however, the rather typical combination of clinical, biochemical and neuroimaging findings may allow targeted genetic analysis in the majority of PCH6 patients.

## Conclusion

Our cases demonstrate that the characteristic neuroradiological abnormalities of PCH6 such as vermis and cerebellar hypoplasia and progressive pontocerebellar atrophy may be missing in some individuals, further expanding the spectrum of *RARS2* mutations. The absence of a typical neuroimaging pattern should not preclude *RARS2* testing. As the nomenclature of PCH6 is misleading, we propose to replace it by *RARS2* mutations*.*

## Abbreviations

CGH: Comparative genomic hybridization
PCH: Pontocerebellar hypoplasia
PCH6: Pontocerebellar hypoplasia type 6
RC: Respiratory chain
